# Comparison of Characteristics between Patients with H7N9 Living in Rural and Urban Areas of Zhejiang Province, China: A Preliminary Report

**DOI:** 10.1371/journal.pone.0093775

**Published:** 2014-04-07

**Authors:** Jimin Sun, Zhenyu Gong, Huakun Lv, Zhiping Chen, Chengliang Chai, Shelan Liu, Feng Ling, Ye Lu, Jian Cai, Zhao Yu, Ziping Miao, Jiangping Ren, Enfu Chen

**Affiliations:** Zhejiang Provincial Center for Disease Control and Prevention, Hang Zhou, China; The University of Tokyo, Japan

## Abstract

A total of 134 cases of H7N9 influenza infection were identified in 12 provinces of China between March 25 and September 31, 2013. Of these, 46 cases occurred in Zhejiang Province. We carried out a preliminary comparison of characteristics between rural and urban H7N9 cases from Zhejiang Province, China. Field investigations were conducted for each confirmed H7N9 case. A standardized questionnaire was used to collect information about demographics, exposure history, clinical signs and symptoms, timelines of medical visits and care after onset of illness. Of the 46 H7N9 cases in Zhejiang Province identified between March 25 and September 31, 2013, there were 16 rural cases and 30 urban cases. Compared to urban cases, there was a higher proportion of females among the rural cases [11/16 (69%) vs. 6/30 (20%), P = 0.001]. Among the rural cases, 14/15 (93%) with available data had a history of recent poultry exposure, which was significantly higher than that among urban cases (64%, *P* = 0.038). More patients from the rural group had a history of breeding poultry compared with those from the urban group [38% (6/16) vs. 10% (3/30), respectively; P = 0.025]. Interestingly, the median number of medical visits of patients from rural areas was higher than that of patients from urban areas (P = 0.046). There was no difference between the two groups in terms of age distribution, fatality rate, incubation period, symptoms, and underlying medical conditions. In conclusion, compared to patients from urban areas, more patients from rural areas were female, had an exposure history, had a history of breeding poultry, and had a higher number of medical visits. These findings indicate that there are different exposure patterns between patients living in rural and urban areas and that more rural cases were infected through backyard poultry breeding.

## Introduction

On March 31 2013, the Chinese Authorities (the National Health and Family Planning Commission, previously the Ministry of Health) announced the identification of a novel influenza A virus infection, A (H7N9), in three people who were seriously ill, from two Chinese provinces [1]. The virus is an avian influenza A reassortant of A (H7N9) from which the hemagglutinin and the neuraminidase genes originate, and A (H9N2) from which the other six gene segments are derived [2], [3].

A total of 134 cases of H7N9 influenza infection were identified in 12 provinces of China between March 25 and September 31, 2013. Zhejiang Province is located in southeast China and is adjacent to Jiangsu Province and Shanghai city where the first case of H7N9 virus was identified. The first case of H7N9 in Zhejiang Province was identified on April 3 and a total of 46 patients, including 16 from rural areas and 30 from urban areas, were reported between March 25 and September 31, 2013 [4]. Rural cases of H7N9 may present with different characteristics because such patients are likely to have lower income, less education, poorer access to medical care, a different lifestyle and different demographics compared to those from urban areas. For example, the overall mortality and incidence rates for malignant neoplasm among urban residents are higher compared to rural residents due to age distribution, socioeconomic status, availability of quality health care, and ethnic differences [5]–[8] . One study on the epidemiological, clinical and viral characteristics of the H7N9 virus has been conducted to date; it reported that 84% (69/82) of patients with H7N9 are urban residents, but the authors did not compare characteristics between patients from rural and urban areas [9]. In this report, we compare the characteristics of 16 rural H7N9 cases with those of 30 urban cases in Zhejiang Province, China.

## Materials and Methods

### Definition of urban and rural cases

According to ‘the diagnosis and treatment programs of human infections with H7N9 virus’ issued by the Chinese Ministry of Health [10], a suspected case is defined as a patient with influenza-like illness including fever, dry cough, headache, muscle ache, generalized malaise, and either positive laboratory confirmation of an influenza A virus, or recent history of exposure to poultry within one week before the onset of symptoms. We considered a suspected case to be a confirmed case if the H7N9 virus was isolated or H7N9 virus RNA was detected by real-time reverse transcription polymerase chain reaction (rRT-PCR) from respiratory specimens of the patient.

An urban case was defined as a patient with confirmed H7N9 infection who lived in an urban area within the two weeks before the onset of illness. Similarly, a rural case was defined as a patient with confirmed H7N9 infection who lived in a rural area within the two weeks before the onset of illness. Urban areas commonly have a high population density and vast manmade structures, and include cities, towns and conurbations. Rural areas, for example, villages, have a low population density and small settlements where farming is a common way of life.

### Laboratory test assays

RNA was extracted from specimens with QIAamp Viral RNA Mini Kit

(Qiagen) according to the manufacturer's instructions, and tested by rRT-PCR as described previously [2], [9]. Additionally, H7N9 virus was isolated from specimens as described previously [2]. These assays were carried out in biosafety level (BSL) 3 facilities at Zhejiang provincial CDC.

### Data collection and analysis

A standardized questionnaire was used to collect information about demographics, exposure history, clinical signs and symptoms, date of onset, date of first medical visit, number of medical visits after onset, date of hospitalization, date of specimen collection, and date of viral infection confirmation. Exposure history included dates, times, frequency and patterns of exposures to poultry or other animals such as swine and wild birds during the two weeks before the onset of illness.

Descriptive statistics were used to analyze the characteristics of H7N9 cases in rural areas. We used Fisher's exact test or Wilcoxon Rank Sum W Test, as appropriate, to compare the characteristics of subgroups of patients from rural and urban areas. The level of significance was set at *P*<0.05. An ethics waiver was granted and authorized under the National Emergent Public Health Events Act. The investigation was exempt from institutional board assessment.

## Results

### Epidemiological data

From March 25 to September 31, 2013, 16 rural and 30 urban cases of H7N9 infection were identified in Zhejiang Province, China. Compared with urban cases, more rural cases were female [11/16 (69%) vs. 6/30 (20%), P = 0.001]. The median age of patients from rural areas was 61.5 years (range, 32–79 years) and that of those from urban areas was 60 years (range, 35–86 years). The difference was not significant according to Wilcoxon Rand Sum W Test of age distribution between two groups (*P* = 0.603). The fatality rate among cases from rural areas was 25% (4/16), which is similar to that among cases from urban areas, 23% (7/30).

**Figure 1:**
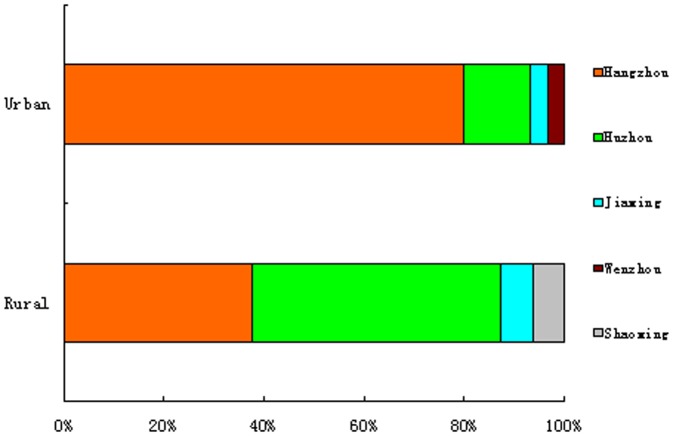
Geographical distribution of patients infected with H7N9 from rural and urban areas of Zhejiang, China.

Cases from rural areas occurred in four districts (Hangzhou, six cases; Huzhou, eight cases; Jiaxing, one case; and Shaoxing, one case), and cases from urban areas also occurred in four districts (Hangzhou, 24 cases; Huzhou, four cases; Jiaxing, one case; Wenzhou one case), as shown in Figure 1. Half of the cases from rural areas occurred in Huzhou.

**Figure 2:**
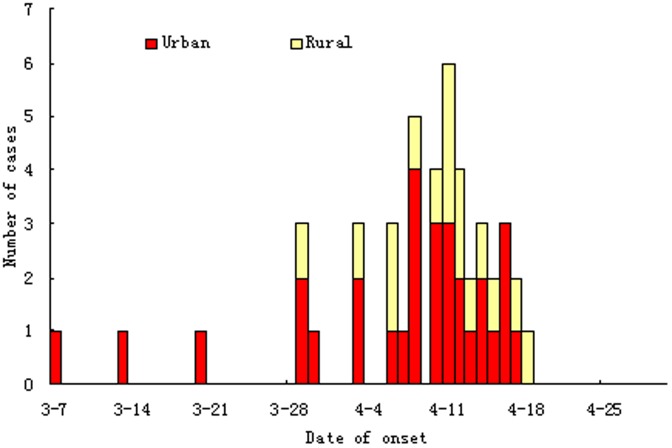
Date of illness onset of patients infected with H7N9 from rural and urban areas.

The first case from an urban area had onset of symptoms on March 7 and the first patient affected from a rural area became ill on March 29. Initially, most cases were from urban areas and then the proportion of patients from rural areas increased (Figure 2).

Among the 16 rural cases, 14 out of 15 with available data had a history of recent poultry exposure, which was significantly higher than that among urban cases [14/15 (93%) vs. 16/25 (64%), P = 0.038]. The type of poultry exposure included chickens, ducks and pigeons, as summarized in Table 1. One rural case and two urban cases had a history of exposure to both chickens and ducks. Types of exposure to poultry in rural and urban cases were reported as: buying poultry (43% vs. 56%), visiting a poultry market (7% vs. 13%), breeding poultry (43% vs. 19%), and killing poultry (31% vs. 6%), respectively. No significant differences were observed between patients from rural areas and those from urban areas in terms of exposure patterns (Table 2). However, compared to all urban cases, more rural cases had a history of breeding poultry [6/16 (38%) vs. 3/30 (10%), P = 0.025]. Of note, three rural cases only bred backyard poultry and did not have a history of exposure to poultry markets.

**Table 1:** Epidemiological characteristics of patients infected with H7N9 from rural and urban areas of Zhejiang, China.

	Characteristics	Rural cases (n = 16)	Urban cases (n = 30)	*P* value
Age and sex	Median age (y)	61.5	60	
	Interquartile range	12.25	32.75	
	Age (20–39)	1 (6%)	7 (23%)	0.603
	Age (40–59)	6 (38%)	8 (27%)	
	Age (60–79)	9 (56%)	13 (43%)	
	Age (80+)	0 (0)	2 (7%)	
	Male	5 (31%)	24 (80%)	0.001
	Female	11 (69%)	6 (20%)	
Exposure history	No poultry exposure (%)	1 (7)	9 (36)	0.038
	Poultry exposure history (%)	14 (93)	16 (64)	
	Chickens (%)	11 (79)	13 (81)	1.000
	Ducks (%)	3 (21)	3 (19)	1.000
	Pigeons (%)	1 (7)	1 (6)	1.000
	Bird (%)	0	1 (6)	
	Exposure frequency			
	Every day before onset (%)	5 (36)	5 (31)	
	<3 days before onset (%)	4 (29)	2 (13)	
	3–7 days before onset (%)	4 (29)	6 (38)	
	>7 days before onset (%)	1 (7)	3 (19)	
	Type of exposure to poultry			
	Buying poultry (%)	6 (43)	9 (56)	0.715
	Visiting poultry market (%)	1 (7)	2 (13)	1.000
	Breeding poultry (%)	6 (43)	3 (19)	0.236
	Killing and cleaning poultry (%)	3 (31)	1 (6)	0.315

**Table 2:** Clinical characteristics of patients infected with H7N9 from rural and urban areas of Zhejiang, China.

	Characteristics	Rural cases (n = 16)	Urban cases (n = 30)	*P* value
Clinical outcome	Hospitalization	16 (100%)	30 (100%)	
	Death	4 (25%)	7 (23%)	0.900
	Survivor	12 (75%)	23 (77%)	
Symptoms at the onset	Fever (%)	16 (100)	30 (100)	
	Cough (%)	12 (75)	25 (83)	0.698
	Expectoration (%)	9 (56)	20 (67)	0.486
	Shivering (%)	4 (25)	8 (27)	1.000
	Fatigue (%)	4 (25)	13 (43)	0.220
	Muscular aches (%)	5 (31)	11 (37)	0.713
	Nausea (%)	1 (6)	2 (7)	1.000
	Vomiting (%)	0	2 (7)	
Underling medical condition	Underlying diseases (%)	11/13 (85)	18/25 (72)	0.386
	Hypertension (%)	8 (73)	10 (56)	0.355
	Diabetes (%)	5 (45)	4 (22)	0.189
	Heart diseases (%)	3 (27)	5 (28)	0.976
	Hepatitis (%)	0	2 (11)	0.252
	Trachitis (%)	1 (9)	3 (17)	0.566
Timelines of medical visits and care (median and interquartile range)	Illness onset to first medical visit	1 (0.25–3)	1.5 (1–3)	0.804
	Illness onset to hospitalization	5 (3–6)	4 (4–6)	0.567
	Illness onset to specimen collection	7 (6–7.75)	5 (4–7.5)	0.091
	Illness onset to confirmation	8 (7.25–9)	6 (5.75–9.25)	0.173
	The number of medical visits	4.5 (4–5)	4 (2–5)	0.046

Of the cases with an exposure history, 93% (13/14) of rural cases and 81% (13/16) of urban cases were exposed to poultry within the seven days before the onset of illness (*P* = 0.602, Table 1). The estimated median incubation period of rural cases and urban cases was 2.5 days and four days, respectively. There were no differences between the two groups in terms of incubation period, onset of illness and symptoms (Table 2).

In 85% (11/13) of rural cases and 72% (18/25) of urban cases with available information the patients had underlying medical conditions (*P* = 0.456). Hypertension was reported in 73% of rural cases (8/11) and 56% of urban cases (10/18) with underlying conditions. Diabetes, heart diseases, trachitis, and hepatitis were also identified in the two groups (Table 2). There was no significant difference in the number of patients with hypertension, diabetes, heart diseases, trachitis, and hepatitis between the two groups.

### Timelines of medical visits and care

Compared with urban cases, the number of medical visits of rural cases was significantly higher, according to a Wilcoxon Rank Sum W Test (P = 0.046). All patients in the rural group and 73% (22/30) of patients in the urban group visited hospital or an outpatient center at least three times. There was no significant difference in duration from illness onset to: first medical visit, hospitalization, specimen collection, and confirmation of infection between the two groups (Table 2).

## Discussion

In our study of urban and rural H7N9 cases in Zhejiang Province, we found that rural cases were more likely to be female, whereas urban cases were more likely to be male. The proportion of cases with exposure history and the median number of medical visits of rural patients were significantly higher than those of urban patients. Compared to urban cases, more rural cases were infected with H7N9 virus through breeding poultry.

A previously published study indicated that 70% of H7N9 cases were male and another study reported that 62% of H7N9 cases in rural areas were male [11]. However, we found that the majority of rural cases were female and that sex distribution was significantly different between the two groups. This might be related to the different habits of rural and urban residents. Generally, in rural areas, female residents go to poultry markets and male residents do the farm work. But, in urban areas, male residents, especially retirees, go to poultry markets. No statistical difference was found in the fatality rate between the two groups, indicating that healthcare utilization might not be a risk factor for fatal outcome.

Two studies have reported that avian influenza A (H7N9) virus can probably be transmitted from person-to-person according to epidemiological investigations; although the transmissibility is limited and non-sustainable [12], [13]. However, in our study, 93% of patients from rural areas with available data had a history of recent poultry exposure and no person-to-person transmission was found. This finding reveals that infected poultry is the principal source of human infections, which is consistent with the result of a previous study [14].

Of interest, the proportion of cases from rural areas with a history of exposure is significantly higher than that of those from urban areas. Although patients from urban areas were less likely to buy poultry from markets, they were probably infected with H7N9 virus in the contaminated environment while visiting the markets. However, we cannot exclude recall bias in this epidemiological investigation.

In China, rural residents usually breed chickens and ducks in their backyards, and these poultry lay eggs before they are slaughtered. They also buy poultry from the market as part of their daily life, and are hence exposed through both breeding and buying poultry. Rural residents could potentially therefore be infected by H7N9 virus through buying poultry in markets or breeding poultry in their backyards. In contrast, the majority of urban residents do not have the means for breeding chickens or ducks, but they tend to buy poultry in markets. Additionally, a greater number of patients from rural areas had an exposure history of breeding poultry than of patients from urban areas. Furthermore, three rural cases were infected through backyard poultry breeding, indicating that poultry in rural areas may have been infected by avian influenza A (H7N9) virus.

H7N9 virus was transmitted in poultry markets in urban areas and no further H7N9 cases were identified after the affected markets were closed. However, H7N9 virus could have been transmitted among backyard poultry in rural areas. Not only were affected poultry markets closed, but it was recommended that backyard poultry should also be slaughtered. H7N9 virus might be transmitted quickly in urban areas because of the higher population density, and this could be the reason for a higher proportion of cases being among urban residents.

Most patients from rural areas presented with flu-like symptoms, such as fever, dry cough, accompanied by headache, muscle aches, and general malaise. The clinical characteristics of H7N9 infection have been reported to be similar among patients from urban areas and in other provinces [15], [16]. The majority of rural cases have an underlying medical background and the proportion is similar to that of urban cases, suggesting that co-morbidities may be a factor for H7N9 infection [17].

There was no difference between urban and rural groups in terms of median time from illness onset to first medical visit, to hospitalization, to a specimen collection, or to confirmation of infection. These findings may be unique to H7N9 because of more awareness among those in rural and urban areas. However, the number of medical visits in the rural group was significantly higher than that of the urban group. This may be related to low income and poor access to medical care in rural areas. Rural residents often receive medical checks in small hospitals or outpatients centers in villages or towns; but these hospitals do not have the capacity to identify and cure H7N9 infection. Patients therefore have to return to receive care in better equipped hospitals, which increases the total number of medical visits among rural cases. The median number of medical visits in rural cases was four; this implies that in some cases there was a delay in both diagnosis and in the golden period for treatment, which can increase the disease severity. It is possible that there are many other infections in patients from rural areas that do not have the opportunity for receiving care in hospitals with the laboratory equipment available to identify the H7N9 virus.

Our study had several limitations. Firstly, we were not able to obtain demographic characteristics of the population in rural areas where H7N9 cases were identified. We were therefore unable to ascertain the reasons for the difference in sex distribution among rural patients. Secondly, we did not research poultry infection in rural areas. We could not confirm whether these poultry were infected with the H7N9 virus. Thirdly, the sample size for rural cases was low. Only 46 patients were identified in Zhejiang Province from April 1 to June 30, 2013. The majority of H7N9 cases in other provinces were in urban residents and we were unable to obtain detailed information on them.

## Conclusions

Compared to the urban group, more patients from the rural group were female, had an exposure history, had a history of breeding poultry, and recorded a higher number of medical visits. These findings indicate that patients from rural areas had different exposure patterns and it is likely that more rural cases were infected via backyard poultry breeding. Further studies, for example to investigate the prevalence of H7N9 among backyard poultry, should be carried out to explore the reasons for different types of exposure to poultry of H7N9 cases in rural areas.
